# Danger to the Eye While Chopping with a Hoe

**Published:** 1900-08

**Authors:** W. L. Bullard

**Affiliations:** Columbus, Ga. (Eye, Ear, Nose and Throat Diseases.) Member of the Georgia State Medical Association ; The American Medical Association, and the Tri-State Medical Association.


### DANGER TO THE EYE WHILE CHOPPING WITH A HOE.

BY W. L. BULLARD, M.D., COLUMBUS, GA.
(EYE, EAR, NOSE AND THROAT DISEASES.)

Member of the Georgia State Medical Association; The American Medical Association, and the Tri-State Medical Association.

Some years ago (vide May, 1885) in the *New York Medical Record* I reported several cases of "Accidents to the Eye While Chopping with the Hoe." Since then I have had not a few similar cases and within the last ten days have had the following three:

J. M. White, aged 26, Jackson's Gap, Ala. Referred to me by the family physician. Patient chopping cotton at the time of accident. Hoe struck a rock and patient "felt some grit strike him in the eye." Continued to work the balance of the day. Next day, on account of pain and redness of eyeball, consulted family physician, who located pieces of steel embedded in the iris, lower portion, nasal side. Patient was referred to me and presented himself on third day after accident. Piece of steel could be seen embedded in the iris, which was contracted, and eyeball considerably injected from corneal and iritic irritation. An operation was advised, which was readily agreed to. The eye was cocainized with a four per cent. solution of cocain, and with the assistance of Dr. McDuffie, of Columbus, Ga., and Dr. Allen, of Oswichee, Ala., I made with a Grafe knife a corneal incision, through which I readily introduced the (Simeon Snell) electro-magnet point, to which the corpus alien adhered and was immediately withdrawn, with prolapse of iris, which was replaced and the eye dressed. Wound healed in a few days and patient returned home with both normal vision and pupil.

CASE 2.—A. H., boy, white, 13 years old, Dadeville, Ala. Chopping with a hoe and struck a rock and "felt some grit strike the eye." Next day consulted the family physician, who advised enucleation, which was objected to. Patient had suffered excruciating pain all night before, and father had gotten up during the night and dropped some castor-oil in the eye "to cool the fever." The following day I was consulted and found the eye in an advanced stage of panophthalmitis. Ball was perforated at corneosclerotic junction, lower portion. A bloody sanguineous discharge was flowing from the ball through the perforation. At first glance eye was known to be lost—irretrievably so. Father was told this and an enucleation advised which was objected to. Electro-magnet was introduced, but foreign body could not be found. Excision again advised, and after one night for deliberation and consultation by "maw" and "paw" (during which time the patient was suffering the tortures of "The Man with the Hoe") an operation was consented to. So, with Dr. A. Floyd, of Phœnix, Ala., I removed the ball, which gave the patient grateful ease, and in three days he was taken to his home in Dadeville, Ala. After enucleation ball was examined, but foreign body was not found. In a few weeks an artificial eye will be fitted.

CASE 3.—A negro girl sent to me by Dr. Young, of Midland, Ga. Patient two days before, while chopping with a hoe, was struck in the right eye by a piece of "grit." Continued to work for two days and consulted Dr. Young, who located the piece of steel embedded in the iris and at once referred the case to me. On examination I found the eye considerably inflamed. I at once cocainized the eye and incised the cornea. The electro-magnet was introduced, and on removal a bit of the hoe, size of a small pinhead, was firmly adhered thereto. The iris prolapsed, which was replaced and the eye bandaged. The next day iris was again found protruding, which at this time was excised. Wound soon healed and patient perfectly satisfied with result—ball saved and vision good.

It will be noticed that almost invariably *hoi polloi* are impressed with the false idea that it is "grit" or pieces of stone that strike and penetrate the ball instead of bits of steel from the hoe.

*June 17th, 1900.*

Since writing the above I have had this additional case:

W. H. C., aged 29, white, married, Tohopeta, Ala. Chopping with a hoe and "felt a piece of grit strike the eye." Continued to work balance of the day. On the following day consulted family physician, who could not discover the foreign body, but gave some "drops to keep the fever down." Eye continued red and inflamed, though patient continued to work for two weeks, at which time I was consulted for enucleation of ball. Found eye red, pupil partially and unevenly dilated, indicative of irido-cyclitis. Cornea clear save an opacity at point of penetration of foreign body. Just beneath this opacity could be seen in the iris a perforation, which, on account of its deception, had been mistaken by several who had examined the eye for the foreign substance. After dilating the pupil as much as possible, the piece of steel could be seen by reflex illumination, behind the iris, at edge of lens, lower portion. After cocainizing the eye an incision was made through the cornea, above the alien corpus, and the electro-magnet point introduced, to which the piece of steel, about the size of a gnat, was readily drawn and extricated from within the eyeball, to the edification of Dr. Cosby and Mr. Albert Allen, a medical student, who kindly assisted me in the operation. The eye was dressed and rapidly progressed to perfect recovery, and patient returned home highly elated over the idea of saving his eyeball, not mentioning its perfect vision.

